# Inguinal hernia surgery learning curves by associate clinicians

**DOI:** 10.1007/s00464-022-09726-5

**Published:** 2022-10-27

**Authors:** Jurre van Kesteren, Pauline J. Meylahn-Jansen, Amara Conteh, Birgit I. Lissenberg-Witte, Alex J. van Duinen, Thomas Ashley, H. Jaap Bonjer, Håkon A. Bolkan

**Affiliations:** 1 Department of Surgery, Amsterdam University Medical Centers, Location Vrije Universiteit, De Boelelaan 1117, 1081 HV Amsterdam, The Netherlands; 2 Global Surgery Amsterdam, Amsterdam, The Netherlands; 3 Department of Internal Medicine, Haaglanden Medical Center, The Hague, The Netherlands; 4 Masanga Hospital, Tonkolili, Sierra Leone; 5 Department of Epidemiology and Data Science, Amsterdam University Medical Centers, Location Vrije Universiteit, Amsterdam, The Netherlands; 6 Institute of Clinical and Molecular Medicine, Norwegian University of Science and Technology (NTNU), Trondheim, Norway; 7 Clinic of Surgery, St. Olavs Hospital HF, Trondheim University Hospital, Trondheim, Norway; 8 CapaCare, Trondheim, Norway; 9 Kamakwie Wesleyan Hospital, Kamakwie, Sierra Leone; 10 Department of General Surgery, North Cumbria University Hospital, Carlisle, UK

**Keywords:** Hernia surgery, Surgical training, Learning curves, Associate clinicians, Task sharing

## Abstract

**Background:**

Open inguinal hernia repair is the most commonly performed procedure in general surgery in sub-Saharan Africa, but data on its learning curve are lacking. This study evaluated the learning curve characteristics to improve surgical training and enable scaling up hernia surgery in low- and middle-income countries.

**Methods:**

Logbook data of associate clinicians enrolled in a surgical training program in Sierra Leone were collected and their first 55 hernia surgeries following the Bassini technique (herniorrhaphies) were analyzed in cohorts of five cases. Studied variables were gradient of decline of operating time, variation in operating time, and length of stay (LOS). Eleven subsequent cohorts of each five herniorrhaphies were investigated.

**Results:**

Seventy-five trainees enrolled in the training program between 2011 and 2020 were eligible for inclusion. Thirty-one (41.3%) performed the minimum of 55 herniorrhaphies, and had also complete personal logbook data. Mean operating times dropped from 79.6 (95% CI 75.3–84.0) to 48.6 (95% CI 44.3–52.9) minutes between the first and last cohort, while standard deviation in operating time nearly halved to 15.4 (95% CI 11.7–20.0) minutes, and LOS was shortened by 3 days (8.5 days, 95%CI 6.1–10.8 *vs.* 5.4 days, 95% 3.1–7.6). Operating times flattened after 31–35 cases which corresponded with 1.5 years of training.

**Conclusions:**

The learning curve of inguinal hernia surgery for associate clinicians flattens after 31–35 procedures. Training programs can be tailored based on this finding. The recorded learning curve may serve as a baseline for future training techniques.

**Supplementary Information:**

The online version contains supplementary material available at 10.1007/s00464-022-09726-5.

To augment the global surgical workforce across all geographical regions and income groups, a rational redistributing of medical tasks between middle cadre health care workers, commonly called associate clinicians (ACs), and medical doctors (MDs) is recommended by the WHO [1, 2]. This restructuring of care is known as task sharing, and regarded safe, cost- and time-effective [3–6] for surgical care in low- and middle-income countries (LMICs), and has led to improved retention of staff at the district level [7]. Surgical task-sharing is practiced in at least 41 LMICs, where approximately 4 out of 10 general surgery procedures are performed by ACs [8].

In 2011 a postgraduate surgical training Program for ACs was initiated by the Sierra Leonean Ministry of Health and Sanitation (MoHS) and the non-governmental organization CapaCare [5]. Sierra Leone is a low-income country in West Africa [9] with an estimated population of 8.3 million [10], and key health indicators on life expectancy, infant-, and under-5 mortality among the poorest in the world [9]. The total surgical workforce consists of 313 full time positions, of which 66 are ACs [11].

Knowledge on the pace of acquiring and mastering surgical skills among ACs is limited. In the context of medical education, learning curves based on serial evaluation of operation-specific outcomes are thought to provide insight about the progress of a trainee [12, 13]. While it was demonstrated that the operating time of ACs decreased significantly during their first 15 cesarean sections and then gradually stabilized [14], yet for other essential surgical procedures, the time until competency is reached is unknown.

Despite recommendations for open inguinal hernia repair to be enforced with a mesh [15–17], costs, availability, and unawareness of mesh use [18], leave the sutured repair *ad modem Bassini* the dominant surgical technique in sub-Saharan Africa [19–22]. In Sierra Leone, sutured groin hernia repair is the most frequently performed operative procedure [23].

The aim of this study was to evaluate the learning curve for non-mesh open inguinal hernia repair *ad modem Bassini*, performed by ACs within a task sharing surgical training program in Sierra Leone. To enhance surgical training and provide objective assessment parameters, the primary goal was to determine the number of inguinal hernia repairs that surgical trainees perform until flattening of operating time was observed. Secondary aims were to evaluate the variation in operating time, and length of hospitalization (LOS).

## Materials and methods

In this prospective cohort study, logbook data from ACs enrolled in a task sharing surgical training program in Sierra Leone were analyzed. The training curriculum was designed to enable ACs to safely manage the most common surgical and obstetric emergencies encountered at first level hospitals and is previously described in detail [5]. Entry requirements for selected ACs (hereinafter referred to as ‘trainee’) were completion of three years of medical training as a clinical officer, and at least two years of clinical working experience. The training program started in 2011, and trainees enrolled biannually. All trainees followed a six-month introductory course which included supervised herniorrhaphy. Subsequently, ACs attended three clinical rotations of each six months in partner hospitals, followed by a one-year internship in a tertiary referral hospital [5].

### Study population

Trainees enrolled in the surgical training between January 2011 and December 2019 were eligible for inclusion. Exclusion criteria were fewer than 55 logged open non-mesh procedures for inguinal hernia using Bassini technique [22] (Supplementary Table), and incomplete personal logbook data. All procedures were supervised and performed in men, aged 18 years or older. Directly supervised is when the trainee performs key components of the procedure with the trainer scrubbed. Indirectly supervised is when the trainee completes the procedure from start to finish and the trainer is unscrubbed. Additional data were collected on type of surgery (planned or emergency), use of performed anesthesia (local, regional, or general), and clinical setting (national referral hospital, regional referral hospital, district hospital or private hospital). The operating time was defined as the time in minutes between the first incision and the last suture of the skin. Logbook data errors in the notation of ante meridiem (am) and post meridiem (pm), leading to operating times lasting approximately 24 h were corrected. Herniorrhaphies that lasted less than 15 min, or more than 180 min were excluded. Exceeding these time margins was considered erroneous in logbook registration, or caused by additional circumstances e.g., herniorrhaphies complicated by bowel perforation. The cut-off times were set by JVK, and two consultant surgeons. All herniorrhaphies were divided into eleven cohorts. The first cohort composed the first five herniorrhaphies of all surgical trainees and the last cohort contained operations 51–55. The number of procedures needed to reach competency was defined as the cohort after which no further statistically significant reduction in operating time was found.

### Sample size estimation

A sample size calculation was performed, and a minimum of 128 operations in each cohort were needed, resulting in a sample size of at least 26 trainees to detect a difference in mean operating time of five minutes (difference to detect) between subsequent cohorts with a power of 80 percent. The standardized difference was calculated by dividing the ‘difference to detect’ by the ‘standard deviation’ (SD). The estimated SD (20 min) was a derivative from learning curve studies by Miyazaki et al. and Harjai et al. both reporting on operating times of the Lichtenstein tension-free mesh inguinal hernia repair [15, 23]. As the Lichtenstein and Bassini procedures are both open inguinal hernia repairs of which operating times are considered rather similar [24], the number of herniorrhaphies included per surgical trainee was based on an analysis of the learning curve of the Lichtenstein. In order not to miss the inflection point at which there is no further significant reduction in operating time, the primary outcomes were evaluated on the means of the first 55 herniorrhaphies. This corresponded to 1.5 times the number of corrected open hernias with mesh, before the inflection point was reached. To assess if the established learning curve was representative for all trainees, a sub-analysis was performed also including the trainees who left the training program prematurely due to insufficient progress, or for personal reasons and thereby failed to perform the required minimum of 55 herniorrhaphies. The sub-analysis on operating times was identically performed as for the group with ≥ 55 herniorrhaphies.

### Data management

Trainees kept record of operation specific data in paper-based logbooks and after each surgery, procedures were checked and signed by the supervising surgeon. The logbook data were transferred to excel and uploaded to an online server hosted by CapaCare and for this study transferred to protected servers hosted by Leiden University Medical Center, Leiden, The Netherlands. Data were checked for missing values and typing errors, and cleaned for duplicates. Logbook data from trainees was excluded from analysis, when ≥ 3 operating times among the first 55 herniorrhaphies were missing. All changes were logged.

### Statistical analysis

Changes in operating time and LOS between subsequent cohorts were compared using a linear mixed model analysis, with random intercept for trainee and a compound symmetry correlation matrix to account for the five different herniorrhaphies in each cohort from the same trainee. Changes in the variation in operating time, computed as the standard deviation of the operating time within each cohort for each trainee separately, were assessed using a linear mixed model as well, with random intercept for trainee. Differences in changes of operating time between trainees with ≥ 55 herniorrhaphies and those with < 55 herniorrhaphies were analyses using the linear mixed models as before, but with group and the two-way interaction between group and cohort added. Data are described by frequency and percentage for categorical variables, and by mean and standard deviation (SD) or median and interquartile range (IQR) for normally and non-normally distributed variables. To assess if the established learning curve was representative for all trainees, a sub-analysis was performed also including the trainees who left the training program prematurely due to insufficient progress, or for personal reasons and thereby failed to perform the required minimum of 55 herniorrhaphies. The sub-analysis on operating times was identically performed as for the group with ≥ 55 herniorrhaphies. All analyses were performed using SPSS statistics version 25 (IBM Corp., Armonk, NY, USA). *p*-values < 0.05 were considered statistically significant.

### Ethics

All patient and trainee data were anonymized. All trainees consented to their anonymized logbook data to be used for research by CapaCare and affiliated researchers. The Masanga Medical Research Unit Scientific Review Committee (SRC-number: MMRU-SRC-007-2019) and the Sierra Leone Ethics and Scientific Review Committee (06/2019) granted approval.

## Results

**Fig. 1:**
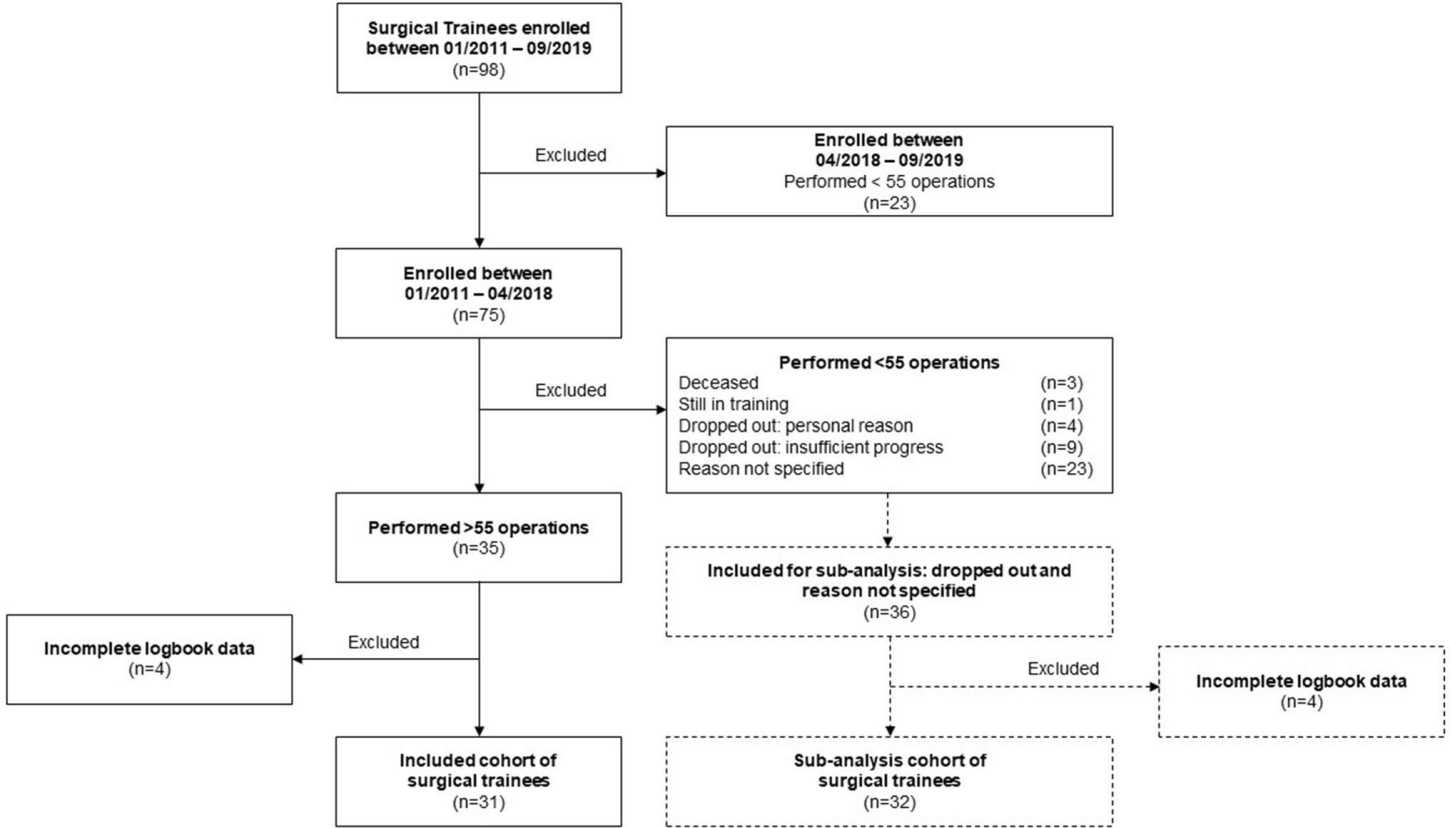
Inclusion of surgical trainees. None of the trainees enrolled after April 2018 was included, as none had performed the required minimum of 55 herniorrhaphies by the 31st of December 2019. Incomplete logbook data were defined as 3 or more missing operating times

In total, 98 trainees enrolled in surgical training between January 2011 and December 2019, logging 54,980 operations, of which 12,030 (21.9%) were herniorrhaphies. On average, trainees observed a median of 2 (IQR 4) and assisted a median of 3 (IQR 4) herniorrhaphies before performing their first hernia procedure under supervision. All trainees enrolled after April 2018 were excluded from this study, as none had performed the required minimum of 55 herniorrhaphies by the 31st of December 2019. From the remaining 75 trainees eligible for inclusion, 31 (41.3%) had complete logbook data and performed each a minimum of 55 herniorrhaphies. Logbook data of 32 (42.7%) trainees that left or had to leave the surgical training program prematurely, or did not perform 55 herniorrhaphies for reasons not specified, were included for the sub-analysis on operating time (Fig. 1).

### Baseline characteristics

Of the 1,705 herniorrhaphies from the 31 trainees who had performed the minimum of 55 herniorrhaphies, 49 (2.9%) am/pm time errors were corrected, 30 (1.8%) operating times exceeded the set time boundaries and were removed, and 11 (0.6%) duplicates were removed. The majority of the included herniorrhaphies were planned procedures (1,609, 95.7%), performed under regional anesthesia (1,085, 64.2%) in a private hospital (1,083, 65.2%) with direct supervision (1,136, 66.6%). During the first two consecutive periods of 6 months in training, 393 (23.1%) and 452 (26.5%) herniorrhaphies were performed, respectively, corresponding to a mean of 12.7 (SD, 6.6) herniorrhaphies per trainee during the first 6 months of training and 14.6 (SD, 9.2) herniorrhaphies during the second 6 months of training. Most trainees had performed 55 herniorrhaphies by the second semester of the second year (Table 1).

**Table 1:** Trainee and patient demographics of all included herniorrhaphies (*n* = 1,705)

Trainee and patient characteristics				
Trainees	*n*	(%)	Mean	(SD)
Number of herniorrhaphies performed				
Year 1: 0–6 months in training	393	(23.0)	12.7	(6.6)
Year 1: 6–12 months in training	452	(26.5)	14.6	(9.2)
Year 2: 12–18 months in training	427	(25.0)	13.8	(10.8)
Year 2: 18–24 months in training	304	(17.8)	9.8	(10.0)
Year 3: 24–30 months in training	44	(2.6)	1.4	(3.2)
Year 3: 30–36 months in training	12	(0.7)	0.4	(1.4)
> 36 months in training	73	(4.3)	2.4	(6.8)
Type of supervision during surgery				
Indirect supervision	569	(33.4)		
Direct supervision	1136	(66.6)		
Training location/hospital*				
National referral hospital	3	(0.2)		
Regional referral hospital	470	(28.3)		
District hospital	105	(6.3)		
Private hospital	1,083	(65.2)		

Patients	*n*	(%)
Age (mean, SD)	43	(16)
Moment of surgery*		
Planned/elective	1609	(95.7)
Emergency procedure	63	(4.3)
Type of anesthesia*		
Local	343	(20.3)
Regional/spinal	1085	(64.2)
General/ketamine	261	(15.5)

All percentages are valid percentages. In the surgical training program, trainees changed clinical internships every 6 months

*Not all numbers round up to 1,705 due to missing logbook data

#Local = lidocaine. Regional = lumbar block (± local anesthesia). General = narcosis or ketamine

### Operating time

The mean herniorrhaphy operating time for the first five procedures was 79.6 min (95% CI 75.3–84.0), and declined to 75.8 min (95% CI 71.4–80.1, *p* = 0.15) for the second cohort. In the subsequent cohorts, a further reduction in mean operating time was observed to 48.6 min (95% CI 44.3–52.9) for the eleventh slot. While a significant reduction in time was found between the third and fourth cohort (66.3 vs. 58.4 min, *p* = 0.003), the decrease in time between cohort six and seven (54.3 vs. 49.2 min, *p* = 0.054) was not significant (Table 2). After the seventh cohort, operations 31 to 35, the mean time of surgery stabilized around 49.2 min.

**Table 2:** Operating time in minutes during the first 55 herniorrhaphies of 31 surgical trainees, and a sub-analysis of 32 surgical trainees with less than 55 herniorrhaphies

Cohort	Herniorrhaphy	≥ 55 Procedures mean operating time	95% CI	*p*-value	< 55 Procedures mean operating time	95% CI	*p*-value
1	1–5	79.6	75.3–84.0	–	89.3	83.8–94.7	–
2	6–10	75.8	71.4–80.1	0.15	78.0	72.3–83.6	< 0.001
3	11–15	66.3	61.9–70.7	< 0.001	69.2	63.2–75.1	0.011
4	16–20	58.4	54.1–62.8	0.003	65.6	59.5–71.7	0.31
5	21–25	59.3	55.0–63.6	0.75	53.7	47.5–59.9	0.001
6	26–30	54.3	50.0–58.7	0.063	54.4	48.0–60.8	0.85
7	31–35	49.2	44.9–53.5	0.054	57.8	51.0–64.7	0.40
8	36–40	50.4	46.1–54.7	0.66	52.6	45.1–60.1	0.24
9	41–45	48.2	43.9–52.6	0.42	54.1	45.7–62.5	0.77
10	46–50	49.8	45.5–54.2	0.54	57.5	47.8–67.1	0.56
11	51–55	48.6	44.3–52.9	0.64	47.5	31.5–63.6	0.27

The *p*-values were based on a linear mixed model analysis, comparing the operating time of each cohort with the previous cohort

**Fig. 2:**
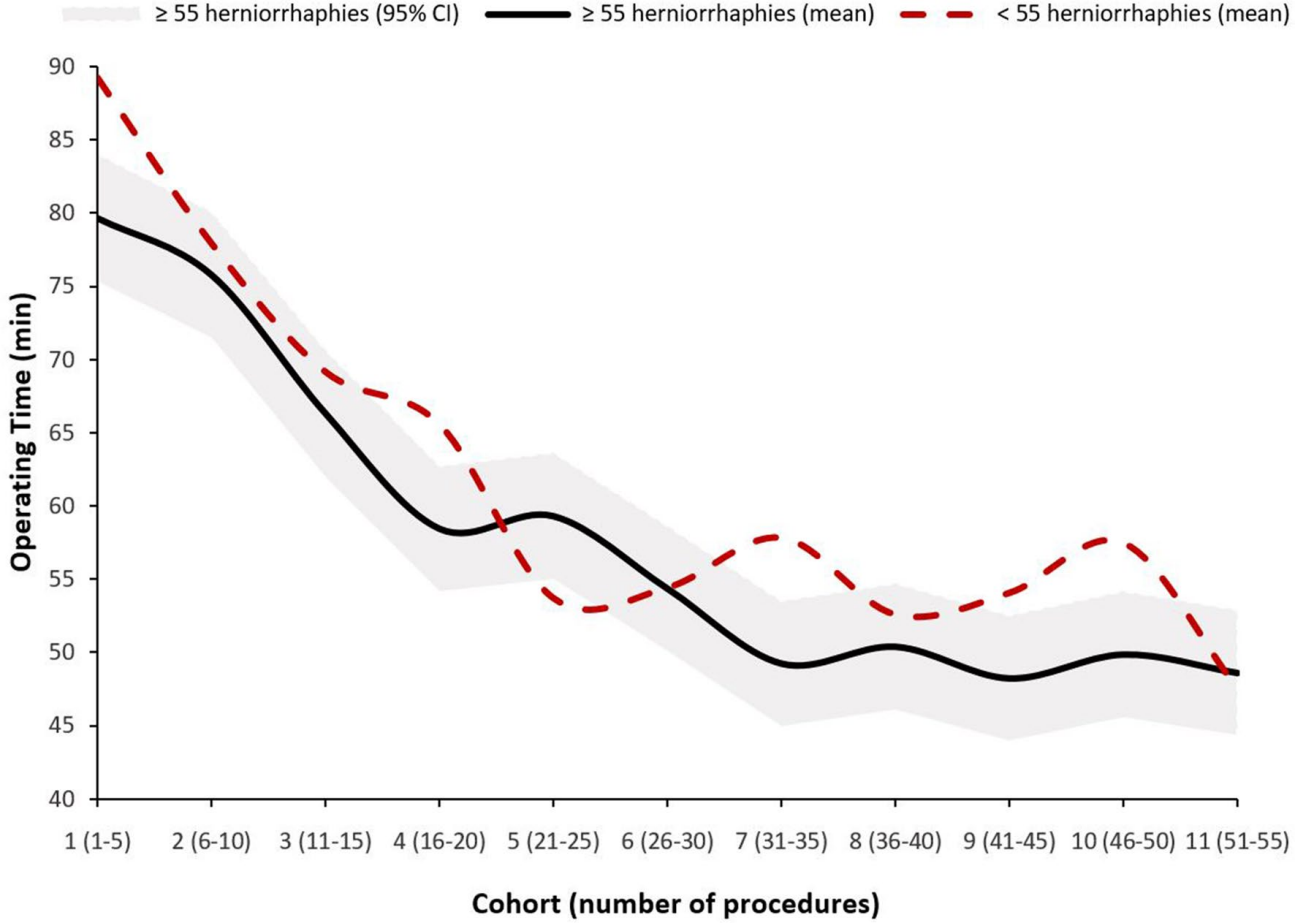
Decline of mean operating times for two groups of trainees. The thick black line visualizes the gradient of decline of the mean operating time for the included (*n* = 31) surgical trainees. The grey area around the black line represents the 95% confidence interval of the operating time in each cohort. The dotted red line visualizes the sub-analysis of 32 surgical trainees that performed fewer than 55 herniorrhaphies. In this graph, the 95% confidence interval is not displayed for this subset of trainees

The sub-analysis for the 32 trainees who had logged less than 55 herniorrhaphies revealed a longer mean operating time for the first cohort; 89.3 min (95% CI 83.8–94.7) but similar operating time of 47.5 min (95% CI 31.5–63.6) for the 11th cohort (Table 2). The total number of included herniorrhaphies per surgical trainee in the sub-analysis varied from two to 54 procedures. Five of 32 trainees had performed between 50 and 54 herniorrhaphies and contributed with data to the 11th cohort. A significant reduction in operating time was found between the fourth and fifth cohort (65.6 vs. 53.7 min, *p* = 0.001). This was not followed by a stabilizing mean time of surgery. Moreover, the evaluation on the trajectory of learning curve was significantly different, compared with trainees with more than 55 herniorrhaphies (*p* = 0.034) (Fig. 2).

### Variation in operating time

The mean SD in operating time per cohort decreased from 26.6 (95% CI 22.8–30.3) to 15.4 (95% CI 11.7–20.0) minutes between the first and last cohort (*p* < 0.001). There were no statistically significant reductions in mean standard deviation observed between consecutive cohorts (Table 3).

**Table 3:** Variation in operating time expressed as mean standard deviation per cohort in minutes during the first 55 herniorrhaphies of 31 surgical trainees

Variation in operating time				
Cohort	Herniorrhaphy	Mean SD (min.)	95% CI	*p*-value
1	1–5	26.6	22.8–30.3	–
2	6–10	23.2	19.5–26.9	0.20
3	11–15	20.0	16.3–23.7	0.23
4	16–20	17.3	13.6–21.0	0.31
5	21–25	19.5	15.8–23.3	0.40
6	26–30	19.1	15.3–22.8	0.86
7	31–35	15.2	11.4–18.9	0.14
8	36–40	16.7	13.0–20.4	0.55
9	41–45	12.1	8.4–15.8	0.08
10	46–50	14.7	11.0–18.4	0.32
11	51–55	15.4	11.7–20.0	0.80

The *p*-values were based on a linear mixed model analysis, comparing the operating time of each cohort with the previous cohort

### Length of stay (LOS)

The length of stay was calculated as a prolonged hospitalization may reflect on complications after surgery, and thereby on the quality of performed procedure. Data on LOS were missing for 159 (9.3%) herniorrhaphies. The mean LOS decreased from 8.5 (95% CI 6.1–10.8) days during the first cohort until 5.4 (95% CI 3.1–7.6) days for patients undergoing the 51–55 herniorrhaphy by the trainees. Despite a downward trend, the overall reduction in length of stay was not statistically significant (*p* = 0.38) (Table 4).

**Table 4:** Length of hospital stay in days during the first 55 herniorrhaphies of 31 surgical trainees

Length of stay				
Cohort	Herniorrhaphy	Days (mean)	95% CI	*p*-value
1	1–5	8.5	6.1–10.8	–
2	6–10	8.9	6.7–11.2	0.77
3	11–15	7.5	5.2–9.7	0.35
4	16–20	7.6	5.4–9.8	0.93
5	21–25	7.5	5.2–9.8	0.94
6	26–30	6.2	3.9–8.5	0.41
7	31–35	6.1	3.8–8.4	0.97
8	36–40	6.6	4.3–8.8	0.77
9	41–45	7.9	5.6–10.1	0.41
10	46–50	5.8	3.5–8.1	0.18
11	51–55	5.4	3.1–7.6	0.81

The *p*-values were based on a linear mixed model analysis, comparing the operating time of each cohort with the previous cohort

## Discussion

This prospective study is the first to evaluate non-mesh herniorrhaphy learning curve characteristics for associate clinicians in a low resource setting. During the first 20 operations, significant reductions in mean operating time were observed. The mean operating time decreased from 79.6 min to 58.4 min for the 4th cohort, before stabilizing around 49.2 min during the 31st—35th herniorrhaphies. This finding was in line with near similarly designed studies that reported greatest decline in operating time early in the learning curve of trainees performing cesarean sections [14], and a cumulative sum control chart (CUSUM) analysis demonstrating similar operating times between surgical residents and surgeons after performing 37—42 tension-free mesh repairs [25]. In addition, we demonstrated that trainees who dropped out, or failed to perform 55 herniorrhaphies during the three years of surgical training, had a significantly different trajectory on the surgical learning curve.

A learning curve is divided into phases, starting with commencement of training, followed by a steep gradient in decline, before only small improvements are observed through additional experience, until achieving stabilization in operating time during the final phase [12, 26]. The estimated time before the plateau phase was reached, corresponded roughly to 1.5 years of surgical training for the associate clinicians enrolled in this study. This finding provides information on minimum requirements for directly supervised operations, while trainee evaluations can be scheduled at set intervals comparing trainees’ personal logbooks with corresponding learning curves. These findings may also help in the design of curricula for surgical training programs in LMICs. Also, this study could function as a baseline-study to assess the effects of future training techniques e.g., simulation-based training to decrease or shorten learning curves [3]. Finally, these findings generated insight into reported learning curve times for the most commonly performed general surgical procedure in sub-Saharan Africa, a condition globally affecting more than 200 million people [27–30].

Strengths of the study include the large prospective data set and design of the analysis, in which intraoperative and postoperative outcomes were evaluated [31, 32]. Relevance is especially pronounced as variation in operating time and reductions in LOS were observed up to the 45^th^ operation, implying that the learning process continued after the found significant reductions in mean operating time and stabilization of operating times.

A recent systematic review of learning curves for minimally invasive abdominal surgery based on 592 included articles, recommended that the ideal learning curve assessment consists of data from different surgeons and hospitals, multiple variables are included and applying a multivariate analysis [26]. All these recommendations were incorporated in this study. In addition, a sample size calculation was performed, something that was only done in 2% of the studies in the review [26]. Our substantial data set on logged herniorrhaphies, allowed for a split in the data into small intervals consisting of five consecutive operations per cohort. Many studies evaluating surgical learning curves divide data into larger cohorts, such as tertials or quartiles. These divisions can compromise the precision of the infliction point on the learning curve [31]. Other studies use more advanced methods but then only examine the learning curve of individuals [25], hindering generalizability among different surgical training programs.

A novelty of this study was the use of variation in operating time as an outcome measure. It was hypothesized that with increasing skill, surgical trainees can better anticipate difficult sections of the procedure and deal with setbacks faster. The assumption is that this may help trainees reflect on anticipation of complications and the skill of the trainee, while also being used for correct planning of operation schedules. It is advised that further studies on variation in operating time also focus on intraoperative variation in individual trainees.

Although this study was conducted among associate clinicians of a surgical training program in a lower resourced setting, the clinical implications may also apply to surgical residents with a medical degree, and peers in higher resourced settings. In Tanzania, the 30-days outcomes after non-obstetric surgical procedures, such as elective groin hernia repairs performed by associate clinicians and MDs were found to be identical [33], and hernia recurrence at 1 year, after Lichtenstein procedure, was recently found to be lower among patients operated by associate clinicians in Sierra Leone [29]. Moreover, the flattened mean operating time of trainees in our study was similar to those procedures performed by surgeons and residents in Canada, Germany and Japan [15, 34]. While earlier research was primarily concerned with recognizing the possibilities of deploying associate clinicians, the field of global surgery has evolved and must now focus on finding solutions to the challenges of scaling-up surgical training programs for associate clinicians, as well as building capacity for elective surgical care in lower resourced settings [29].

One of the limitations of this study, is the method of data collection. All collected data came from self-maintained paper-based individual logbooks. While the requirement of supervising surgeons signing the trainees’ logbooks after each procedure, may have contributed to integrity of the data, the quality of the data could have been improved using an electronic logbook [35]. Furthermore, observations during surgery, data on recurrences, and better registration on the postoperative outcomes infections, neuralgia and seroma could have completed a competence assessment of surgical trainees, while the recurrence rate could have been an indication on the quality of herniorrhaphies [15, 16, 23, 36]. In light of this missing observational data, a median caseload of 64 (range 18–110) inguinal hernia repairs have previously been described for surgical trainees in the UK to achieve the level to perform the procedure unsupervised and be able to deal with complications [13]. Procedure based assessments were included to demonstrate competency, yet this was not part of this study.

The reduction in LOS from 8.5 to 5.4 days should be interpreted with caution as this may have been caused by differences in hospital policies. While hernia surgery is often a day-care procedure in high resourced settings [37], similar LOS outcomes were found in other lower resourced settings and should therefore be interpreted in this context [15, 23, 36, 38, 39]. Lengthier admission in lower resourced settings may be caused by the severity and size of inguinal hernias [20, 21], but was also linked to cultural and insurance-technical arguments [15]. However, shorter LOS could increase surgical capacity of overstretched health facilities in lower resourced settings. Studies from Nigeria and Sierra Leone showed that outpatient hernia surgery was feasible without adverse effects on patients [29, 40]. As the trainees of this study enrolled over a nine-year timespan, it is unlikely that the trend towards shorter hospital admissions over the years can fully explain our findings. Each of the eleven cohorts that made up the learning curve, contained surgeries that were performed at the beginning, and at the end of the inclusion period.

Lastly, the 31 included trainees with 55 operations are likely a selected group of the best students, referring to the different learning curve trajectory compared to the 32 drop-outs. Furthermore, the exclusion criteria of this study could have led to an underestimation of the number of herniorrhaphies needed to become proficient. Cases of strangulated hernias, which are generally more challenging, were excluded from this study.

This prospective cohort study examined the learning curve for non-mesh open inguinal hernia repair for associate clinicians in a surgical training program in West Africa. Flattening of operating time was reached after performing 31–35 herniorrhaphies. Reduction in the variation of operating time and length of hospitalization continued beyond this point in training. The results of this study are recommended for use in improving surgical training programs in lower resourced settings as well as to serve as a baseline to evaluate innovative surgical training techniques.

## Supplementary Information

Below is the link to the electronic supplementary material.

Supplementary file1 (DOCX 15 KB)
