# Supplementary material for: Inguinal hernia surgery learning curves by associate clinicians
Source: Surg Endosc. 2022 Oct 27;37(3):2085–94. doi: 10.1007/s00464-022-09726-5 (PMC10017565; doi:10.1007/s00464-022-09726-5)
Supplement: Supplementary file 1 — Supplementary file1 (DOCX 15 KB) [file 464_2022_9726_MOESM1_ESM.docx]

**Supplementary table**

| **Herniorrhaphy steps *ad modum* Bassini** |
| --- |
| Incision in the inguinal region in a skin crease 1–2 cm above the inguinal ligament |
| Divide and ligate superficial inferior epigastric vein |
| Incise through Scarpa’s fascia and the external oblique aponeurosis |
| Lift and retract edges of aponeurosis while extending incision |
| Identification and protection of the ilioinguinal nerve |
| Identification and mobilization of the spermatic cord |
| Free the cord from the hernia sac and dissect the sack until the neck |
| *If* direct: Hernia sack pushed back and posterior wall reinforced with absorbable suture |
| *If* indirect: Suture ligate the neck after ensuring sac is empty. Reinforce posterior wall with interrupted non-absorbable suture (e.g., No. 1 Nylon); starting medially at the pubic tubercle. Insert next stitches through conjoined tendon and inguinal ligament and progress laterally |
| Replace ilioinguinal nerve |
| Hemostasis if required |
| Close external oblique and Scarpa’s fascia with continuous 2/0 absorbable suture |
| Close the skin with interrupted 2/0 suture and place a dressing |

**Supplementary table** The technique described in this table is for the repair of inguinal hernias in men. The aim of the operation is to reduce the hernia, ligate the sac and repair the defect in the posterior inguinal canal. All steps were derived from the World Health Organization textbook *Surgical Care at the District Hospital*. This textbook is part of the standard training curriculum for trainees in the CapaCare Surgical Training Program in Sierra Leone.
